# Variation in lipid synthesis, but genetic homogeneity, among *Leptopilina* parasitic wasp populations

**DOI:** 10.1002/ece3.4265

**Published:** 2018-06-27

**Authors:** Bertanne Visser, Thierry Hance, Christine Noël, Christophe Pels, Masahito T. Kimura, Johannes Stökl, Elzemiek Geuverink, Caroline M. Nieberding

**Affiliations:** ^1^ Biodiversity Research Centre (ELIB) Earth and Life Institute (ELI) Université catholique de Louvain Louvain‐la‐Neuve Belgium; ^2^ Hokkaido University Museum Hokkaido University Sapporo Japan; ^3^ Institute of Insect Biotechnology Justus‐Liebig‐University Gießen Gießen Germany; ^4^ Groningen Institute for Evolutionary Life Sciences University of Groningen Groningen the Netherlands

**Keywords:** coevolution, *Drosophila*, Hymenoptera, molecular markers, parasitoids

## Abstract

Lipid synthesis can have a major effect on survival and reproduction, yet most insect parasitoids fail to synthesize lipids. For parasitic wasps in the genus *Leptopilina,* however*,* studies have suggested that there is intraspecific variation in the ability for lipid synthesis. These studies were performed on only few populations, and a large‐scale investigation of both lipogenic ability and population genetic structure is now needed. Here, we first examined lipogenic ability of nine *Leptopilina heterotom*a populations collected in 2013 and found that five of nine populations synthesized lipids. The 2013 populations could not be used to determine genetic structure; hence, we obtained another 20 populations in 2016 that were tested for lipogenic ability. Thirteen of 20 populations (all *Leptopilina heterotom*a) were then used to determine the level of genetic differentiation (i.e., haplotype and nucleotide diversity) by sequencing neutral mitochondrial (*COI*) and nuclear (ITS2) markers. None of the 2016 populations synthesized lipids, and no genetic differentiation was found. Our results did reveal a nearly twofold increase in mean wasp lipid content at emergence in populations obtained in 2016 compared to 2013. We propose that our results can be explained by plasticity in lipid synthesis, where lipogenic ability is determined by environmental factors, such as developmental temperature and/or the amount of lipids carried over from the host.

## 1 INTRODUCTION

The ability of animals to store energy reserves in the form of fat is essential for both survival and reproduction (Arrese & Soulages, 2010; Hazel, 1995; Turkish & Sturley, 2009). Storage fat can help overcome harsh environmental conditions, such as times at which food is not available, which is an all‐pervasive challenge for many animals (McCue, Terblanche, & Benoit, 2017). Numerous insects, for example, can survive long periods without food, such as diapause, by accumulating large lipid reserves for use during winter when foraging is impossible (Hahn & Denlinger, 2011). Lipids are also a critical component of the egg in oviparous animals (Geister, Lorenz, Hoffmann, & Fischer, 2008; Sloggett & Lorenz, 2008; Sotherland & Rahn, 1987), constituting approximately 30%–40% of total macronutrients in insect eggs (Muller et al., 2017). Lipids can further serve as an important energetic substrate fueling flight (Arrese & Soulages, 2010; Kemp & Alcock, 2003; Zera, Sall, & Otto, 1999). The amount of storage lipids available throughout life can thus have major fitness effects, and lipid synthesis is a highly conserved traits (Ballard, Melvin, & Simpson, 2008; Jakob, Marshall, & Uetz, 1993; Kemp & Alcock, 2003). Animals thus generally start accumulating fat for storage as a reserve when a surplus of food is available (Birsoy, Festuccia, & Laplante, 2013; Wakil, 1989).

Unlike many other animals, several insect parasitoids were found to lack the ability for lipid synthesis. These insects fail to synthesize storage lipids following sugar‐feeding, which typically stimulates lipid synthesis (Visser & Ellers, 2008). Parasitoids have a parasitic larval lifestyle, where development is spent feeding in or on an arthropod host (Godfray, 1994). The ability for lipid synthesis was lost repeatedly during the evolution of distinct parasitoid taxa, including beetles, flies, and wasps, as a consequence of the parasitic larval lifestyle (Visser et al., 2010). Parasitoid larvae can readily consume the lipid stores of their host, suggesting that lipid synthesis in parasitoids is redundant or even costly to maintain (Visser, Willett, Harvey, & Alborn, 2017). While the majority of parasitoids lack the ability for lipid synthesis, several phylogenetically distinct taxa were found capable of lipid synthesis (Visser et al., 2010). As the lack of lipid synthesis was found to be ancestral in parasitic hymenoptera, lipid synthesis seems to have re‐evolved independently in some parasitic wasp species.

**Figure 1:**
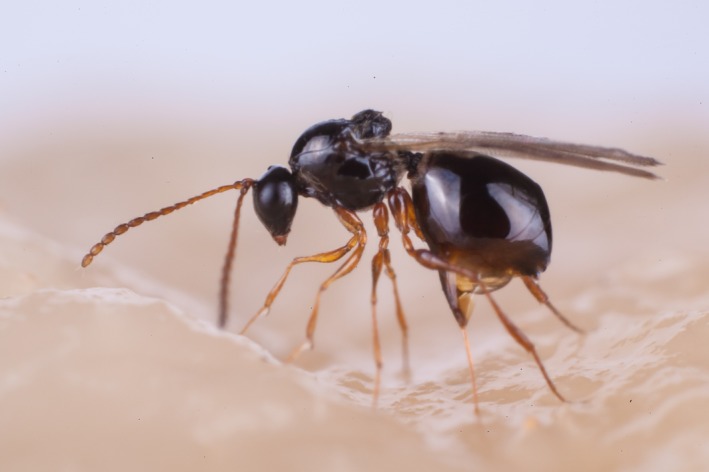
Model parasitic wasp *Leptopilina heterotoma*. Photograph courtesy of Hans Smid from BugsinthePicture, http://www.bugsinthepicture.nl

Between‐species variation in the ability for lipid synthesis became evident by testing a large number of taxonomically distinct parasitoid species (Visser et al., 2010), but only few species were tested repeatedly for the ability to synthesize lipids (Giron & Casas, 2003; Rivero & West, 2002; Visser et al., 2012, 2017). An exception are species in the genus *Leptopilina*, which have been popular model systems for a multitude of research fields, including, but not limited to, studies on (theoretical) ecology and behavior (e.g., foraging behavior), chemical communication (e.g., host‐finding cues), life histories (e.g., time vs egg limitation), and physiology (e.g., host immunity) (Fleury, Gibert, Ris, & Allemand, 2009; Haccou, Vlas, Alphen, & Visser, 1991; Heavner et al., 2017; Janssen, van Alphen, Sabelis, & Bakker, 1995; Visser, van Alphen, & Hemerik, 1992; Wertheim, Vet, & Dicke, 2003). Initially, *L. heterotoma* (Figure 1) was found to lack lipid synthesis (Eijs, Ellers, & van Duinen, 1998), but data on another population later revealed active lipid synthesis (Le Lann et al., 2014; Visser et al., 2010). In a study using the closely related species *Leptopilina boulardi*, four populations were tested using the same host species that revealed contrasting lipogenic phenotypes: two populations synthesized lipids, while two populations did not (Moiroux et al., 2010). Later work on these same four populations then revealed a strong genetic structure with populations synthesizing lipids being genetically closer to each other than to populations that lacked lipid synthesis (Seyahooei, van Alphen, & Kraaijeveld, 2011). These results suggest that genetic divergence corresponds to the observed variation in ability for lipid synthesis in *L. boulardi* populations.

A large‐scale investigation of both the ability for lipid synthesis and population genetic structure (haplotype and nucleotide diversity) in *Leptopilina* wasps is now needed. Here, we started by collecting nine different *L. heterotoma* populations from the field in Europe in 2013 and tested these populations for the ability to synthesize lipids. Based on previous results in *Leptopilina* (Eijs et al., 1998; Le Lann et al., 2014; Moiroux et al., 2010; Visser et al., 2010)*,* we expected to find variation in the ability for lipid synthesis between populations. Intraspecific variation in ability for lipid synthesis was indeed observed between these populations, but all nine cultures perished before genetic structure could be determined. In a renewed effort, a total of 20 populations from Europe and Asia were then obtained from other laboratories or the field in 2016: 19 populations belonging to three *Leptopilina* species (*L. heterotoma n* = 13 populations; *L. boulardi n* = 4 populations; and *L. victoriae n* = 2 populations), and one population of a closely related species, *Ganaspis brasiliensis* (Hymenoptera: Figitidae). The latter species is phylogenetically close to *Leptopilina,* and a potential biocontrol agent against the pest *Drosophila suzukii*, which has not yet been tested for lipogenic ability. We then established the genetic structure (including measures of haplotype and nucleotide diversity) of all 13 *L. heterotoma* populations obtained in 2016 by sequencing the mitochondrial *COI* gene and the nuclear Internal Transcribed Spacer 2 (*ITS2*) gene region to quantify genetic divergence between populations. While we predicted to observe variation and genetic differentiation between these *Leptopilina* populations/species, none of the 20 populations tested were found to synthesize lipids and virtually no genetic differentiation was found between the 13 *L. heterotoma* populations. We discuss how differences between the 2013 and 2016 populations can be explained.

## 2 MATERIALS AND METHODS

### 2.1 Insects

In 2013, *Drosophila melanogaster* (Diptera: Drosophilidae) hosts were obtained from a culture collected in Dwingeloo, the Netherlands (see Supporting information Table S1 for GPS coordinates). Hosts were maintained in flasks with continuous access to food medium (20 g agar, 35 g yeast, 50 g sugar, 5 ml nipagin containing 100 g 4‐methyl hydroxyl benzoate in 1L 96% alcohol, and 5 ml propionic acid per liter water) that was replaced every 3–4 days at a temperature of 20^°^C, a relative humidity of 75%, and a photoperiod of L:D 16:8. In 2016, *D. melanogaster* were obtained from an existing laboratory culture that was originally collected in Sainte‐Foy‐les‐Lyon in France in 1994. Hosts were maintained in cages with continuous access to food medium at a temperature of 24°C, a relative humidity of 30%, and a photoperiod of L:D 16:8.

Nine *L. heterotoma* (Hymenoptera: Figitidae) populations obtained in 2013 were collected from the field (see Supporting information Table S1 for GPS coordinates of collection sites) and reared on *D. melanogaster*. *L. heterotoma* females were offered approximately 200 2nd–3rd *D*. *melanogaster* larvae to maintain cultures at a temperature of 20^◦^C, a relative humidity of 75%, and a photoperiod of L:D 16:8. In 2016, 20 populations belonging to the species *Leptopilina heterotoma, L. boulardi, L*. *victoriae,* and *Ganaspis brasiliensis* (Hymenoptera: Figitidae (Nomano et al., 2017)) were obtained from existing laboratory cultures or collected from the field (Supporting information Table S1). Wasp cultures were maintained at a temperature of 23°C, a relative humidity of 75%, and a photoperiod of L:D 16:8. We choose to increase the rearing temperature of wasps in 2016 to be able to maintain populations from all geographic areas (i.e., all populations obtained from other laboratory were already maintained at 23°C).

### 2.2 Testing for lipogenic ability

To test whether wasps synthesize lipids, we conducted feeding experiments similar to those performed in previous studies (Eijs et al., 1998; Le Lann et al., 2014; Moiroux et al., 2010; Visser et al., 2010, 2012). Using this method, a comparison is made between the total amount of storage lipids present right after emergence from the host, that is, teneral lipid levels, and the amount of lipids after feeding on a sugar source (up to 14 days). Lipid extractions were performed using gravimetry as described in Visser et al. (Visser et al., 2010), with the exception that individuals were dried in an oven at 60°C for 3 days before and after extraction of lipids rather than freeze‐dried. Lipid levels were then calculated by subtracting the lipid‐free dry mass from the lipid‐containing dry mass, after which the percentage fat was calculated. In 2013, only females were tested, but in 2016, males were used, because females were used for maintaining cultures of all populations. Although females are typically larger and contain more lipid reserves, there was no a priori assumption that the ability for lipid synthesis would differ between the sexes. To indeed verify that sex did not affect lipogenic ability, similar experiments were performed with females of three of the 2016 *L. heterotoma* populations (Leiden, the Netherlands; Wilsele, Belgium; Eupen, Belgium; Table 1).

**Table 1:** Results of feeding experiments for individuals obtained in 2013 (A) and 2016 (B)

Species	Population	Sex	Mean % fat at emergence ± 1 *SE*	*n*	Mean % fat after feeding ± 1 *SE*	*n*	Test statistic (F or W^a^)	*p*‐value	Lipogenesis?
(A)									
*L. heterotoma*	Dwingeloo (NL)	Females	16.52 ± 1.32	23	17.43 ± 0.59	19	181^a^	0.356	No
*L. heterotoma*	Tiendeveen (NL)	Females	16.87 ± 1.15	21	20.60 ± 0.89	17	−2.463	0.019	Yes
*L. heterotoma*	Rhenen (NL)	Females	16.85 ± 1.00	20	19.16 ± 0.73	17	−1.803	0.08	No
*L. heterotoma*	*Eupen (BE)*	*Females*	*17.16 ± 1.09*	*24*	*21.13 ± 0.97*	*17*	*−2.576*	*0.014*	*Yes*
*L. heterotoma*	Chaudfontaine (BE)	Females	19.54 ± 0.85	18	17.26 ± 0.93	21	1.784	0.083	No
*L. heterotoma*	Haltern (DE)	Females	15.07 ± 1.00	14	21.69 ± 1.52	14	−3.629	0.001	Yes
*L. heterotoma*	Sankt Goar (DE)	Females	12.91 ± 0.81	24	20.88 ± 1.43	7	−4.699	<0.0001	Yes
*L. heterotoma*	Vouvray (FR)	Females	14.35 ± 1.45	22	18.37 ± 1.43	20	−1.973	0.055	No
*L. heterotoma*	Macon (FR)	Females	14.56 ± 1.16	19	20.00 ± 1.56	15	63^a^	0.006	Yes
(B)									
*L. heterotoma*	Vosbergen (NL)	Males	23.50 ± 0.56	29	12.95 ± 0.59	25	165.3	<0.0001	No
*L. heterotoma*	Leiden (NL)	Males	24.56 ± 1.55	19	7.45 ± 0.81	19	342^a^	<0.0001	No
		Females	27.93 ± 1.09	18	13.91 ± 0.58	18	153.6	<0.0001	No
*L. heterotoma*	Wilsele (BE)	Males	24.62 ± 1.05	20	12.96 ± 2.05	18	323^a^	<0.0001	No
		Females	30.61 ± 1.34	20	24.23 ± 1.24	20	12.16	0.001	No
*L. heterotoma*	*Eupen (BE)*	*Males*	*27.20 ± 1.77*	*21*	*12.83 ± 2.62*	*16*	*298* a	*<0.0001*	*No*
		*Females*	*24.92 ± 1.37*	*18*	*23.43 ± 2.20*	*17*	*0.95*	*0.337*	*No*
*L. heterotoma*	St. Ethienne sur Chalaronne (FR)	Males	23.73 ± 0.83	29	7.96 ± 0.42	28	812^a^	<0.0001	No
*L. heterotoma*	Cailloux sur Fontaine (FR)	Males	26.62 ± 0.45	29	10.22 ± 0.36	29	804.8	<0.0001	No
*L. heterotoma*	St. Marcel les Valence (FR)	Males	34.48 ± 1.33	26	14.55 ± 0.633	25	647^a^	<0.0001	No
*L*. *heterotoma*	Bellegarde (FR)	Males	24.79 ± 0.55	29	10.30 ± 0.87	28	783^a^	<0.0001	No
*L. heterotoma*	Santa Christina d'Aro (ES)	Males	25.59 ± 0.87	30	17.05 ± 1.97	29	688^a^	<0.0001	No
*L. heterotoma*	Unkown (DE)	Males	27.05 ± 0.99	28	17.60 ± 1.15	27	668^a^	<0.0001	No
*L. heterotoma*	Whittlesworth (UK)	Males	27.09 ± 1.06	32	10.31 ± 0.55	34	1055^a^	<0.0001	No
*L. heterotoma*	Great Shelford (UK)	Males	24.97 ± 1.01	29	12.57 ± 2.18	26	680^a^	<0.0001	No
*L. heterotoma*	Sapporo (JP)	Males	26.13 ± 1.11	30	35.18 ± 4.60	25	300^a^	0.208	No
*L. boulardi*	St. Foy les Lyon (FR)	Males	29.17 ± 0.84	23	8.57 ± 0.90	17	272.3	<0.0001	No
*L. boulardi*	Avignon (FR)	Males	32.80 ± 1.19	25	14.89 ± 1.90	22	510^a^	<0.0001	No
*L. boulardi*	St. Marcel les Valence (FR)	Males	26.45 ± 1.18	21	9.00 ± 1.11	19	125.5	<0.0001	No
*L. boulardi*	Girona (ES)	Males	29.47 ± 1.13	25	10.08 ± 1.58	18	427^a^	<0.0001	No
*L. victoriae*	Unknown (?)	Males	25.47 ± 1.01	29	6.77 ± 0.53	26	724^a^	<0.0001	No
*L. victoriae*	Kota Kinabalu (MY)	Males	23.77 ± 0.89	29	7.50 ± 0.87	17	146	<0.0001	No
*G. brasiliensis*	Kaohsiung (TW)	Males	24.77 ± 1.00	20	8.62 ± 0.49	21	400^a^	<0.0001	No

Notes.

Populations obtained from similar location: Eupen (presented in italics).

a Test statistic of nonparametric Mann–Whitney *U*‐tests.

### 2.3 Statistics

We are primarily interested in testing whether lipid synthesis occurs within populations; hence one‐way ANOVAs or Mann‐Whitney *U*‐tests (in case of non‐normal data/heterogeneity of variances) were performed for each population separately. A significant increase in lipid levels after sugar‐feeding suggests that lipid synthesis has occurred, whereas lipid synthesis is lacking when lipid levels remain stable or decrease (Eijs et al., 1998; Ellers, 1996; Visser et al., 2010, 2012). We further compared teneral lipid content of female wasps obtained in 2013 and 2016, and between 2013 populations synthesizing and lacking lipid synthesis, to determine whether and when host lipid content may affect lipogenic ability of wasps using one‐way ANOVAs. Statistics were performed using R project version 3.4.1 (R Development Core Team, 2016).

### 2.4 Genetic structure of *L. heterotoma* populations

DNA extraction—Total DNA was extracted from two to five adult males for each of the thirteen *L. heterotoma* populations using the Cetyl Trimethyl Ammonium Bromide (CTAB) extraction method [described in (Navajas, Lagnel, Gutierrez, & Boursot, 1998)]. In short, each male was snap‐frozen in liquid nitrogen and crushed with a plastic pestle in a 1.5‐ml microcentrifuge tube. Two hundred μl of extraction buffer (2% CTAB, 1.4 M NaCl, 0.2% 2‐b mercapto‐ethanol, 20 mM EDTA, 100 mM TRIS‐HCL, pH 8.0, 65°C) and 4 μl protein kinase K (10 mg/ml) were then added, after which samples were incubated at 65°C for 1 hr. Proteins were then removed by adding 200 μl of chloroform/isoamyl alcohol (24/1) and DNA precipitated by adding one volume of isopropanol. Samples were then rinsed with ethanol (76% v/v ethanol containing 10 mM ammonium acetate) and resuspended in 20 μl ultra‐pure water. Two microliters RNase (100 μg/ml) was then added and samples incubated at 37°C during 30 min.

PCR amplification and sequencing—Two partial DNA fragments of the *COI* gene and *ITS2* DNA region were amplified and sequenced. Amplification reactions were performed using a total volume of 15 μl containing 0.125 μl of Taq polymerase (5 U/μl; Roche), 1.5 μl enzyme buffer containing 15 mM MgCl_2_, 0.75 μl of each primer (10 μM), 1.2 μl dNTP (2.5 mM), 9.675 μl water, and 1 μl of DNA. We used the following *COI* and *ITS2* primers: COI‐LCO 5′‐GGTCAACAAATCATAAAGATATTGG‐3′COI‐HCO 5′‐TAAACTTCAGGGTGACCAAAAAATCA‐3′(Folmer, Black, Hoeh, Lutz, & Vrijenhoek, 1994) and ITS2U 5′‐TGTGAACTGCAGGACACATG‐3′ (Campbell, Steffen‐Campbell, & Werren, 1994) ITS2L 5′‐AATGCTTAAATTTAGGGGGTA‐3′ (Schilthuizen, Nordlander, Stouthamer, & van Alphen, 1998). Amplifications were performed using a Veriti Thermal Cycler (Applied Biosystems) with an initial denaturation step at 94°C for 2 min, followed by 35 cycles with 30 s at 94°C, 30 s at 48°C, and 1 min at 72°C with a final extension cycle of 10 min at 72°C for *COI*. For *ITS2,* we used an initial denaturation step at 94°C for 2 min, followed by 35 cycles with 30 s at 94°C, 30 s at 59°C, and 1 min at 72°C with a final extension cycle of 7 min at 72°C. Ten microliters of PCR product purified with Illustra ExoProstar (GE Healthcare) was prepared and send out for sequencing in both directions (3730*xl* DNA Analyzer; Macrogen Inc., Amsterdam). Sequences were aligned, after which consensus sequences were generated using Geneious^®^ software version 10.0.9 (Kearse et al., 2012). Consensus sequences of the two DNA regions were obtained for individuals of all populations, with the exception of *ITS2* for two French populations (Cailloux sur Fontaine, France and Saint Marcel les Valence, France; Table 2). Sequences are available on Genbank: accession numbers MG561215–MG561267. DnaSP software (v. 5(Librado & Rozas, 2009)) was used to calculate nucleotide diversity (*π*) and haplotype diversity (*h*; Table 2). The K_2_P genetic distance was calculated using MEGA software (v. 6(Tamura, Stecher, Peterson, Filipski, & Kumar, 2013)). Median‐joining haplotype networks of *COI* and *ITS2* were generated with PopART (http://popart.otago.ac.nz).

**Table 2:** Nucleotide variation, haplotype number, haplotype diversity (*h*), and nucleotide diversity (*π*) for COI and ITS2 of 13 *Leptopilina heterotom*a populations obtained in 2016

Population code	Individual	COI gene						
		Position				Haplotype no.	*h* (±*SD*)	*π* (±*SD*)
		284	353	446	644			
Vosbergen (NL)	1	T	C	G	A	1	0.000	0.000
	2	T	C	G	A			
Leiden (NL)	1	T	C	G	A	1	0.000	0.000
	2	T	C	G	A			
Wilsele (BE)	1	T	C	G	A	1	0.000	0.000
	2	T	C	G	A			
Eupen (BE)	1	T	C	G	A	1	0.000	0.000
	2	T	C	G	A			
Saint Ethienne sur Chalaronne (FR)	1	T	C	A	A	1	0.000	0.000
	2	NA	NA	NA	NA			
	3	T	C	A	A			
	4	T	C	A	A			
	5	T	C	A	A			
Cailloux sur Fontaine (FR)	1	T	C	G	A	1	0.000	0.000
	2	T	C	G	A			
Saint Marcel les Valence (FR)	1	C	C	G	A	2	1.000 ± 0.500	0.0015 ± 0.0007
	2	T	C	G	A			
Bellegarde (FR)	1	T	C	G	A	1	0.000	0.000
	2	T	C	G	A			
Santa Christina d'Aro (ES)	1	T	C	G	A	1	0.000	0.000
	2	T	C	G	A			
Unkown (DE)	1	T	C	G	A	1	0.000	0.000
	2	T	C	G	A			
Whittlesworth (UK)	1	T	C	G	A	1	0.000	0.000
	2	T	C	G	A			
Great Shelford (UK)	1	T	T	G	A	2	1.000 ± 0.500	0.0015 ± 0.0007
	2	T	C	G	A			
Sapporo (JP)	1	T	C	G	G	1	0.000	0.000
	2	T	C	G	G			

Population code	Individual	*ITS2 region*						
		Position			Haplotype no.	*h* (±*SD*)	*π* (±*SD*)	
		404	405	516				
Vosbergen (NL)	1	—	—	T	1	0.000	0.000	
	2	—	—	T				
Leiden (NL)	1	—	—	T	1	0.000	0.000	
	2	—	—	T				
Wilsele (BE)	1	A	A	—	1	0.000	0.000	
	2	A	A	—				
Eupen (BE)	1	—	—	T	1	0.000	0.000	
	2	—	—	T				
Saint Ethienne sur Chalaronne (FR)	1	A	A	T	1	0.000	0.000	
	2	A	A	T				
	3	A	A	T				
	4	A	A	T				
	5	A	A	T				
Cailloux sur Fontaine (FR)	1	NA	NA	NA				
	2	NA	NA	NA				
Saint Marcel les Valence (FR)	1	NA	NA	NA				
	2	NA	NA	NA				
Bellegarde (FR)	1	—	—	T	1	0.000	0.000	
	2	—	—	T				
Santa Christina d'Aro (ES)	1	—	—	T	1	0.000	0.000	
	2	—	—	T				
Unkown (DE)	1	—	—	T	1	0.000	0.000	
	2	—	—	T				
Whittlesworth (UK)	1	—	—	T	1	0.000	0.000	
	2	—	—	T				
Great Shelford (UK)	1	—	—	T	1	0.000	0.000	
	2	—	—	T				
Sapporo (JP)	1	—	—	T	1	0.000	0.000	
	2	—	—	T				

## 3 RESULTS

### 3.1 Lipogenic ability

**Figure 2:**
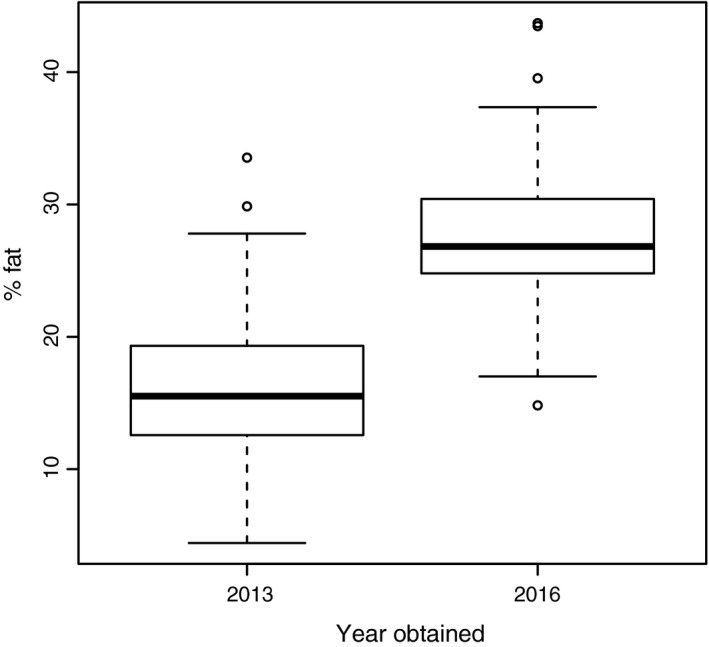
Boxplot showing the median, interquartile range, minimum, and maximum percentage fat at emergence for female *L. heterotoma* wasps collected in 2013 and 2016 (*n* = 241 individuals)

Lipid synthesis varied between populations obtained in 2013. Five of nine populations increased lipid levels, whereas lipid levels remained stable or decreased in the other four populations (Table 1). This is in stark contrast with findings for the 2016 populations, where none of the populations were found to synthesize lipids, including one population that was collected at the same location both years (Table 1). Mean lipid levels of females obtained in 2013 and 2016 differed almost twofold: 2013 females emerged with ~16% fat (±0.4, 1 *SE*), whereas 2016 females emerged with ~28% (±0.8, 1 *SE*) fat (Figure 2). The 2013 populations thus emerged with significantly fewer lipids compared to the 2016 populations (*n* = 241; *F*_1,239 _= 203,5; *p* < 0.0001; Figure 2). Teneral lipid levels (at emergence) were, however, similar between 2013 populations lacking and synthesizing lipids, that is, ~17% (±0.6, 1 *SE*) and ~15% (±0.5, 1 *SE*) respectively (*n* = 185; *F*_1,183 _= 2.915; *p*‐value = 0.0895).

### 3.2 Genetic diversity and structure of *L. heterotoma* populations

**Figure 3:**
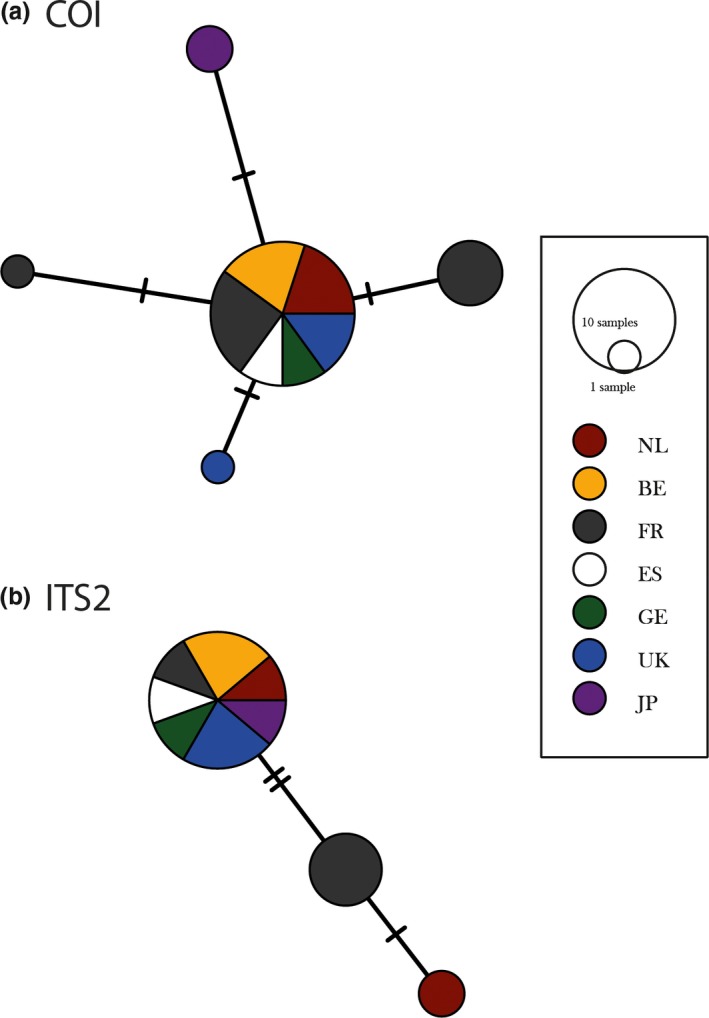
Median‐joining haplotype networks for COI (a) and ITS2 (b) of 13 *Leptopilina heterotoma* populations. Each sample is a single individual with networks clustered by population

*COI* and *ITS2* sequences of thirteen *L. heterotoma* populations obtained in 2016 had an aligned length of 698 and 577 bp, respectively. Populations showed very limited polymorphism (four polymorphic sites for *COI*, 3 polymorphic sites for *ITS2*; Figure 3; Table 2). K_2_P genetic distances ranged between 0 and 0.003 for *COI* with an average of 0.001 (±0.00005, 1 *SE*) over all individuals. For *ITS2* K_2_P distances ranged between 0 and 0.006, with an average for all individuals of 0.002 (±0.0001, 1 *SE*). A median joining network revealed that the Japanese population displays a specific haplotype not shared with any of the other populations for *COI*, but not for *ITS2* (Figure 2). Samples from the French populations were found to be most diverse compared to samples of the other populations for *COI,* but this could be due to the higher representation of French populations (i.e., 4 of 13).

## 4 DISCUSSION

Early comparative work on parasitoids led to the idea that the ability for lipid synthesis in parasitic wasps was lost as an adaptation to the parasitic lifestyle, and that lipid synthesis was a discrete trait, that is, a wasp species either synthesizes lipids or it does not (Visser et al., 2010). Lipid synthesis was then found to vary intraspecifically in the parasitic wasp genus *Leptopilina*, but only one or few populations were ever tested simultaneously (Eijs et al., 1998; Le Lann et al., 2014; Moiroux et al., 2010; Visser et al., 2010). To gain a better understanding of intraspecific variation in lipid metabolism of parasitic wasps, a large‐scale analysis of lipid synthesis in *Leptopilina* was thus needed. We initially confirmed previous findings, as lipid synthesis was found to vary between *L. heterotoma* populations obtained in 2013. Populations obtained in 2016, however, showed contrasting results, where none of the populations from four different parasitic hymenopteran species were shown to synthesize lipids. Moreover, we did not find any genetic differentiation between thirteen *L. heterotoma* populations obtained in 2016, neither for *COI* nor for *ITS2* markers.

Sequence analyses of the neutral markers *COI* and *ITS2* revealed little genetic polymorphism of, and pervasive gene flow, between all thirteen *L. heterotoma* populations. A phylogenetic study by Novkovic, Mitsui, Suwito, and Kimura (2011) revealed divergence between *L. heterotoma* from different localities in Japan for *COI*, but unlike our findings, the *COI* sequence of a population collected in France matched with the one collected in Japan (i.e., Sapporo, from which our Japanese population also originated). In another phylogenetic study*,* little sequence divergence was found between *L. heterotoma* originating from France and the Netherlands, but here only a single individual was sampled per population and only three populations were compared (Schilthuizen et al., 1998). These authors suggested that studying the biogeography of *Drosophila* parasitoids, including *Leptopilina*, is hampered by the potential human‐assisted colonization of new geographic areas. This particularly applies to *L. heterotoma,* a generalist that has been found on most continents (Nordlander, 1980) and is in line with estimates of genetic divergence in *D. melanogaster* (Schlotterer & Tautz, 1994). Man‐made re‐introduction of *L. heterotoma* could thus lead to genetic mixing, diminishing genetic divergence. Our results indeed suggest there is a high level of genetic mixing among populations from geographically distinct areas. Hence, the absence of genetic differentiation among populations in our study suggests that genetic evolution is not involved in explaining the differences across years in ability for lipid synthesis of *Leptopilina* populations.

We propose two alternative mechanisms to explain the discrepancy between our results. First, a comparison of wasp lipid levels at emergence between the 2013 and 2016 populations revealed a nearly twofold difference, with teneral lipid levels (i.e., at emergence) being significantly, and overall twice higher, in the 2016 populations. The 2013 and 2016 populations were reared on two different *D. melanogaster* strains; hence, differences in lipid levels of newly emerged parasitoid adults may be due either to differences in lipid quantities between host strains, or differences in the ability of wasps to carry over lipid reserves. These data indeed suggest that lipid synthesis is an environmentally induced trait in *Leptopilina*, where lipid synthesis is plastic and dependent on the quantity of lipids carried over from the host, such that lipid synthesis is shut down when large lipid stores can be carried over from the host, and activated when hosts contain little fat reserves. Another environmental factor that may affect the plastic induction of lipid synthesis is temperature, because the temperature at which experiments were performed differed between populations collected in 2013 and 2016. Only one study has so far tested the same wasp population at different temperatures (Le Lann et al., 2014): *L. heterotoma* females developed on the same *D. melanogaster* host strain at 20 and 23°C, after which adults were allowed to feed during 7 days. Body size and teneral lipid content did not differ between developmental temperatures, with the latter being ~20% (Le Lann et al., 2014). Results obtained at 20°C, where an increase in lipid levels after feeding was found, were indeed similar to earlier findings (Visser et al., 2010), where the same population, host strain, and temperature were used. Lipids levels remained stable, however, at 23°C (Le Lann et al., 2014). These findings differ from our current results at 23°C, because all populations significantly decreased lipids during life (with the exception of only two populations; Table 1). Temperature may thus interact with host strain to affect lipogenic phenotypes in wasps. In conclusion, we propose that our data on the genetic structure and lipid synthesis of *Leptopilina* populations are best explained by the idea that lipid synthesis is an environmentally induced trait, which could apply also to other parasitic wasp species.

If the induction of lipid synthesis is indeed plastic and dependent on host lipid levels, the propensity to synthesize lipids could vary to a large extent depending on the specific combination of host strain and wasp species tested. Ideally, we would have tested *Leptopilina* species and strains that had already been tested previously (Eijs et al., 1998; Le Lann et al., 2014; Moiroux et al., 2010; Visser et al., 2010). Unfortunately, none of these original strains were available (because most were collected/maintained between 10 and 30 years ago). We also did not have sufficient funding at the time to collect new *L. heterotoma* populations from the same 2013 field locations. There was, however, one exception: a population collected in Eupen, Belgium. In 2013, females of this population emerged with ~17% (±1, 1 *SE*) fat, which increased to ~21% (±1, 1 *SE*) following sugar feeding. In contrast, females of the 2016 population emerged with 27% (±1.4, 1 *SE*) fat, which declined to ~13% (±2.2, 1 *SE*) fat after feeding. While there was a significant increase in lipid levels after feeding for 2013 females, 2016 females had much higher teneral lipid reserves and lacked lipid synthesis. This adds strength to the argument that host strain and host lipid availability play a critical role in determining lipid synthesis of wasps. When taking a closer look at mean teneral lipid levels of all 2013 populations, there seem to be only minor (and non‐significant) differences: ~17% (±0.6, 1 *SE*) for populations lacking lipid synthesis and ~15% (±0.5, 1 *SE*) for populations synthesizing lipids. A comparison with previous data on *L. boulardi* and *L. heterotoma* (Visser et al., 2010) reported teneral lipid levels of 26% (±0.6, 1 *SE*) and 23% (±0.9, 1 *SE*), respectively, where the former was found to lack lipid synthesis, and the latter was found to synthesize lipids. These *L. boulardi* and *L. heterotoma* strains were reared on the same *D. melanogaster* host strain (but a different strain from those used here). Overall, female *L. heterotoma* wasps thus seem to lack lipid synthesis when teneral lipid content lies between ~14% (±1.5, 1 *SE*) (population from Vouvray, France) and 31% (±1.3, 1 *SE*) (population from Wilsele, Belgium), but start synthesizing lipids when teneral lipid levels are between ~13% (±0.8, 1 *SE*) (population from Sankt Goar, Germany) and 23% (±0.9, 1 *SE*; see findings of Visser et al., 2010). We now need to explicitly test when and how host lipid content affects lipogenic ability in parasitic wasps.

## AUTHOR CONTRIBUTIONS

BV and CMN conceived the ideas; BV and CMN designed the experiments. TH, MTK, JS, and EG provided materials and resources. BV, CN, CP, and EG performed fieldwork, experiments, and analyses. BV wrote the manuscript. TH, CN, CP, MTK, JS, EG, and C.M.N. edited the manuscript. BV and CMN acquired the funding.

## DATA ACCESSIBILITY

Data will be made available as supporting information Data S1. DNA sequences: Genbank accessions MG561215 – MG561267 **.**
